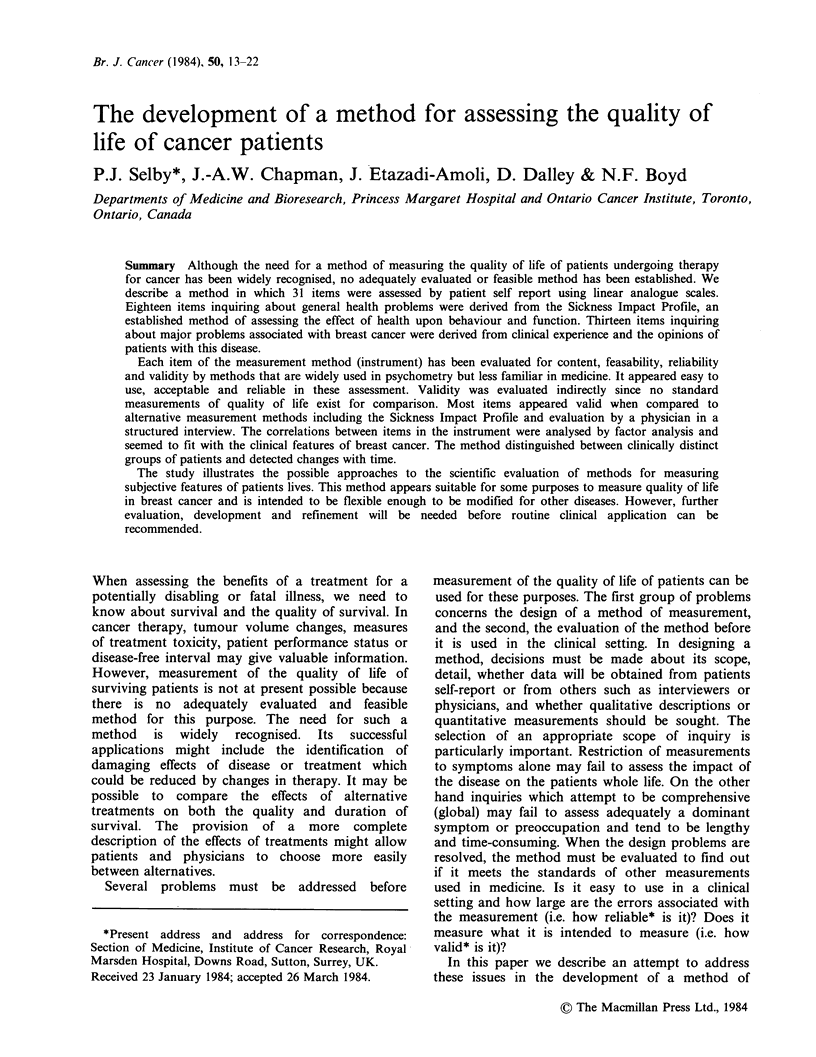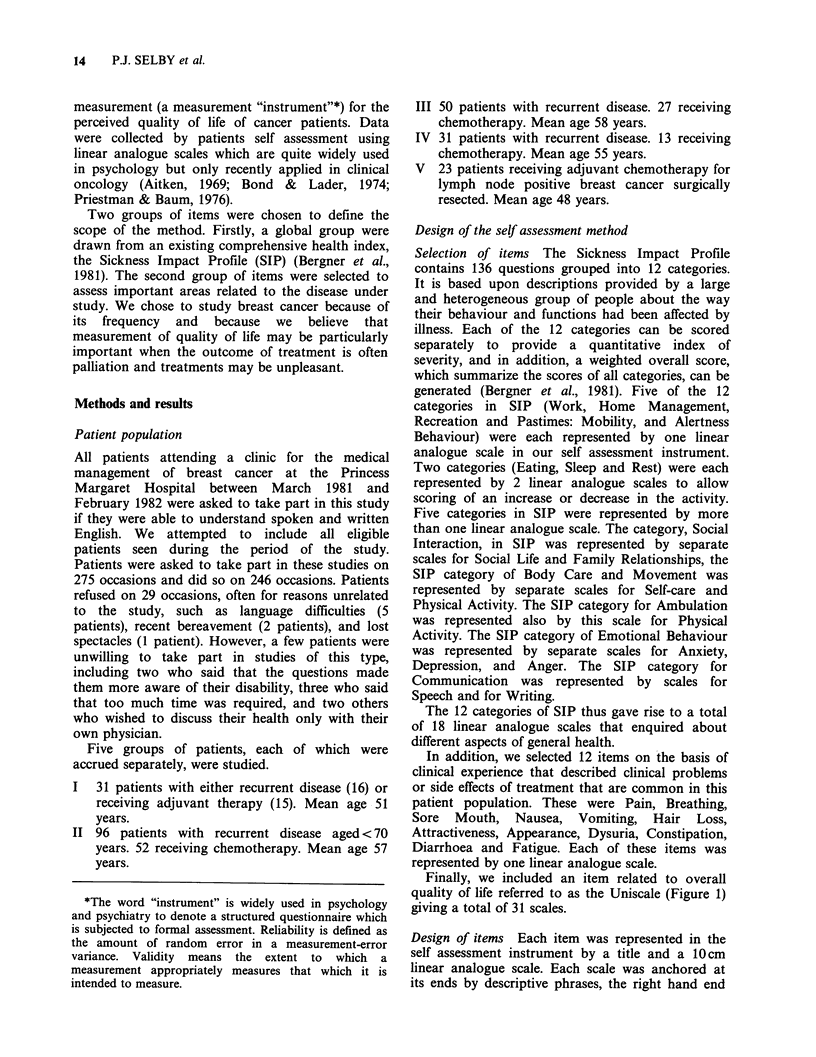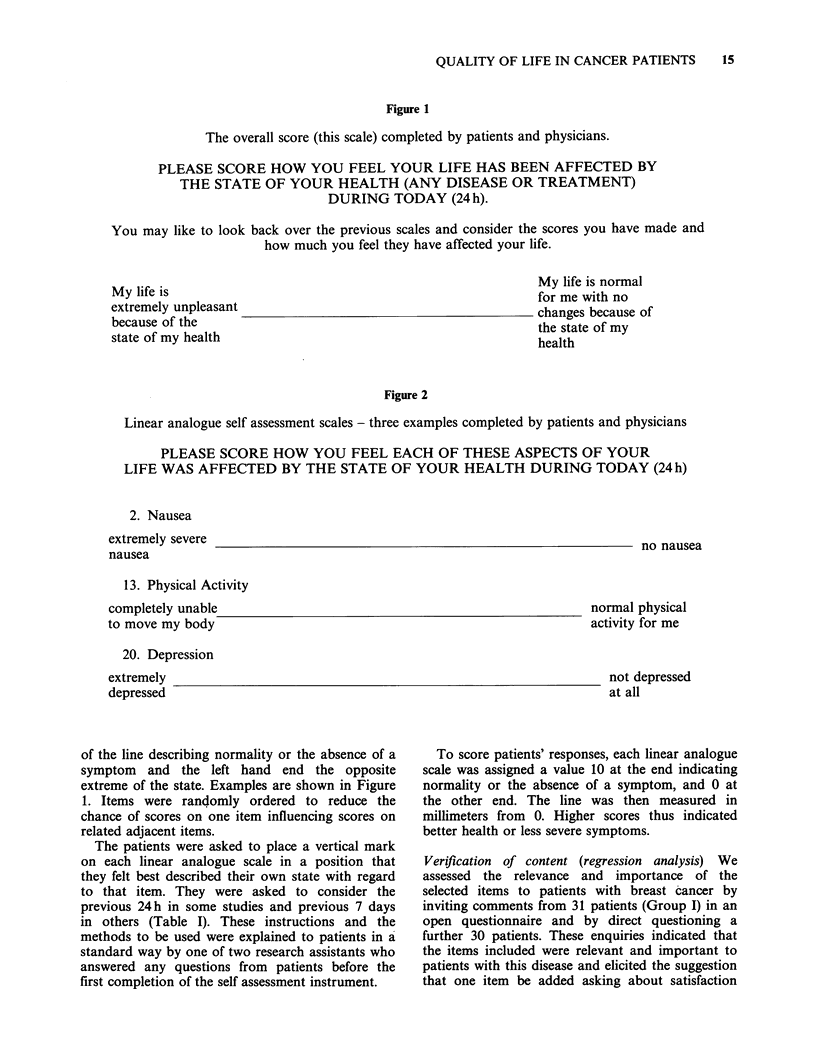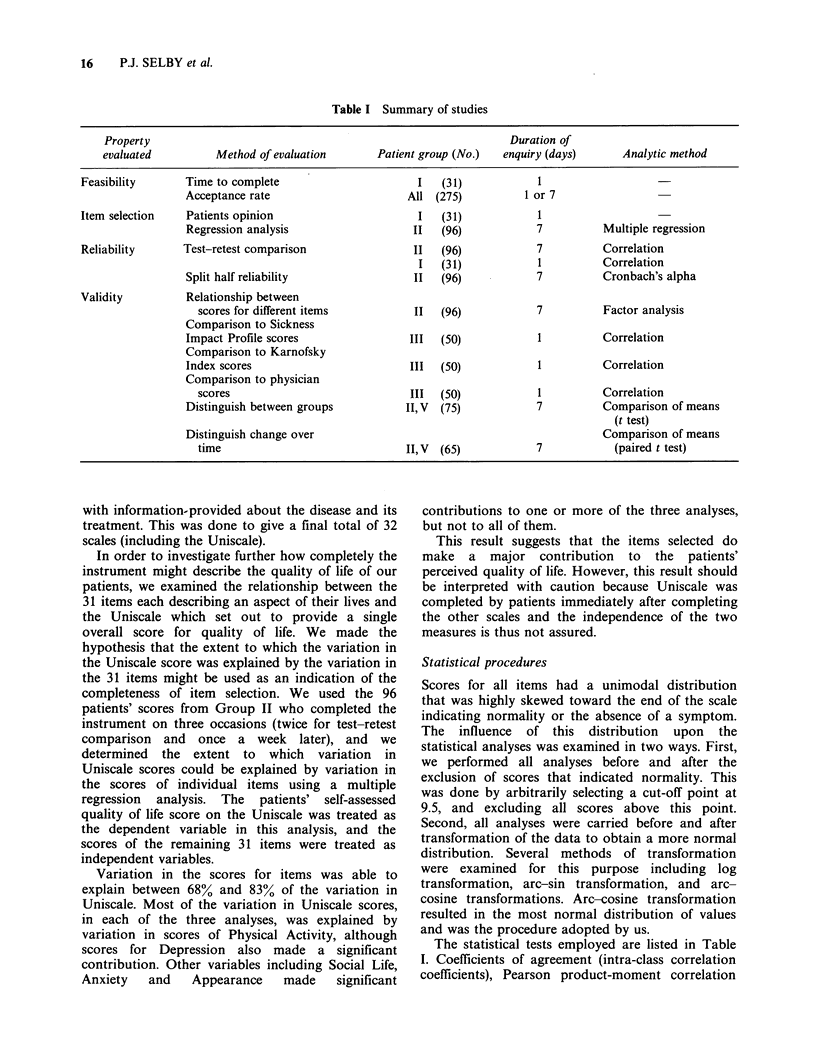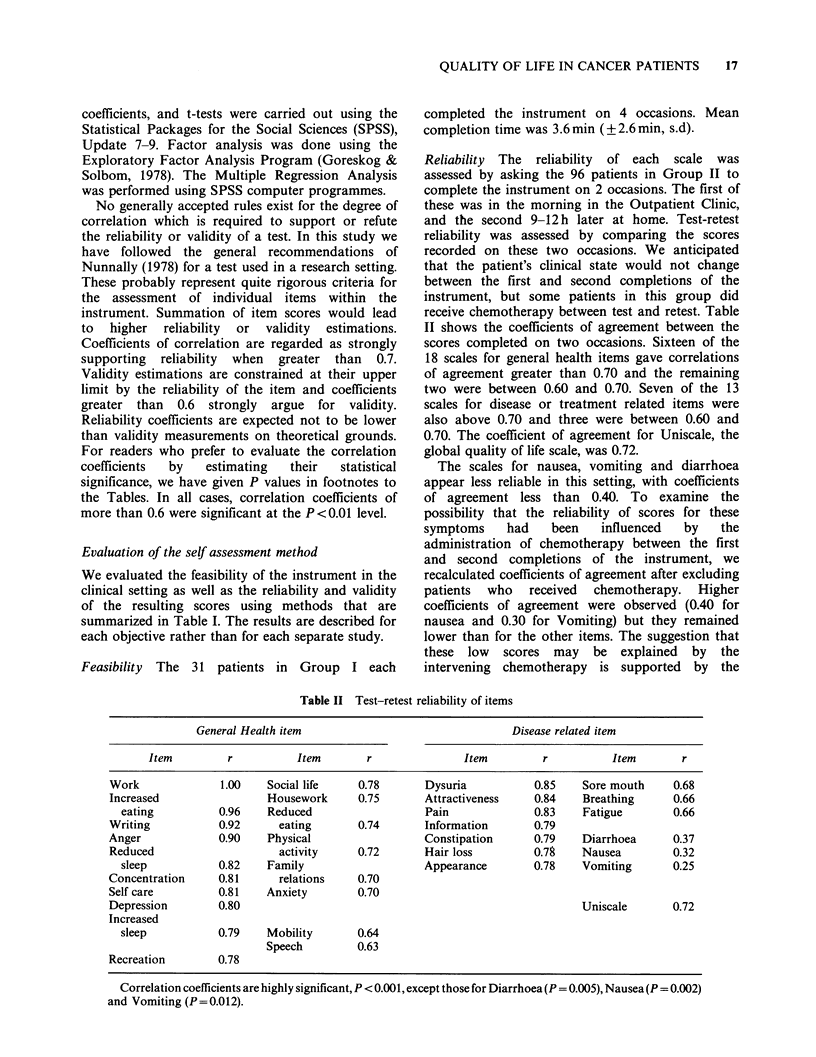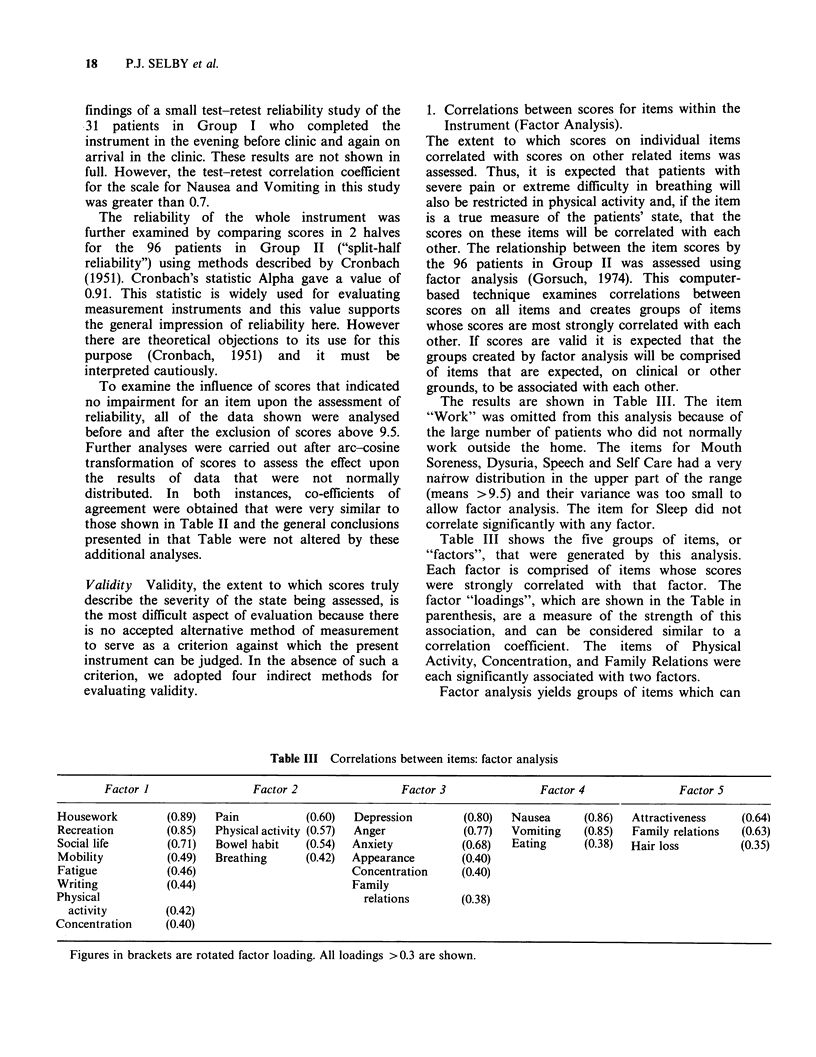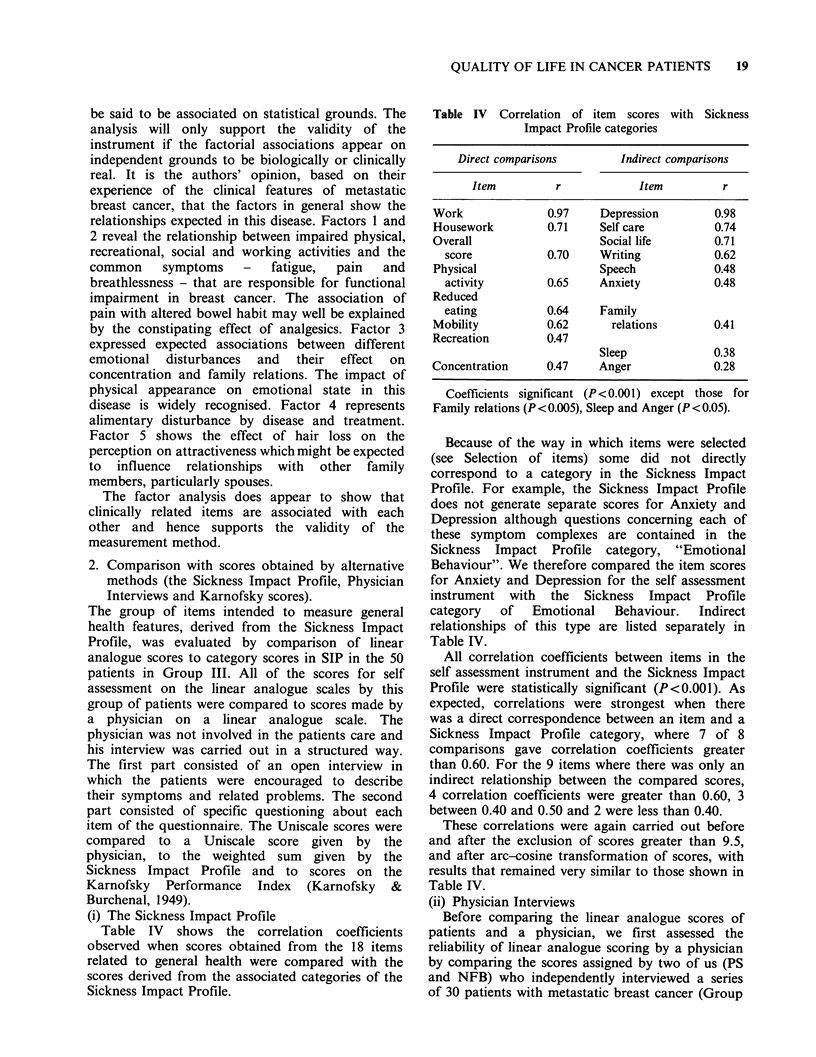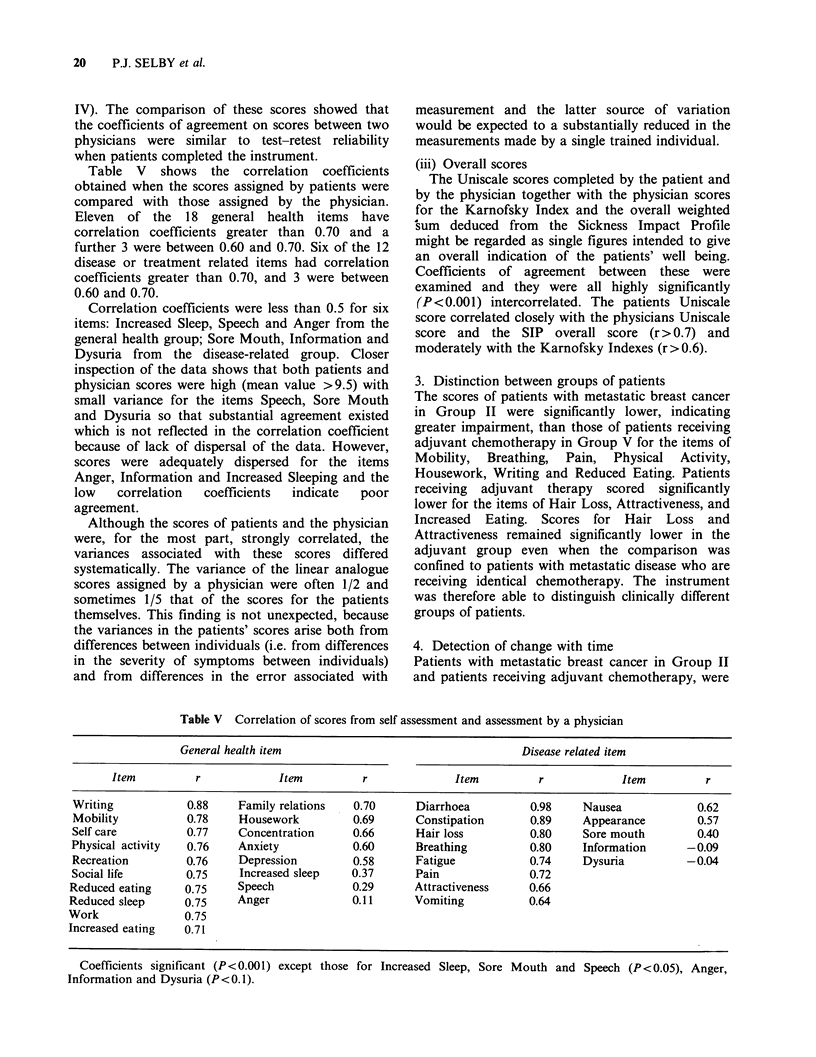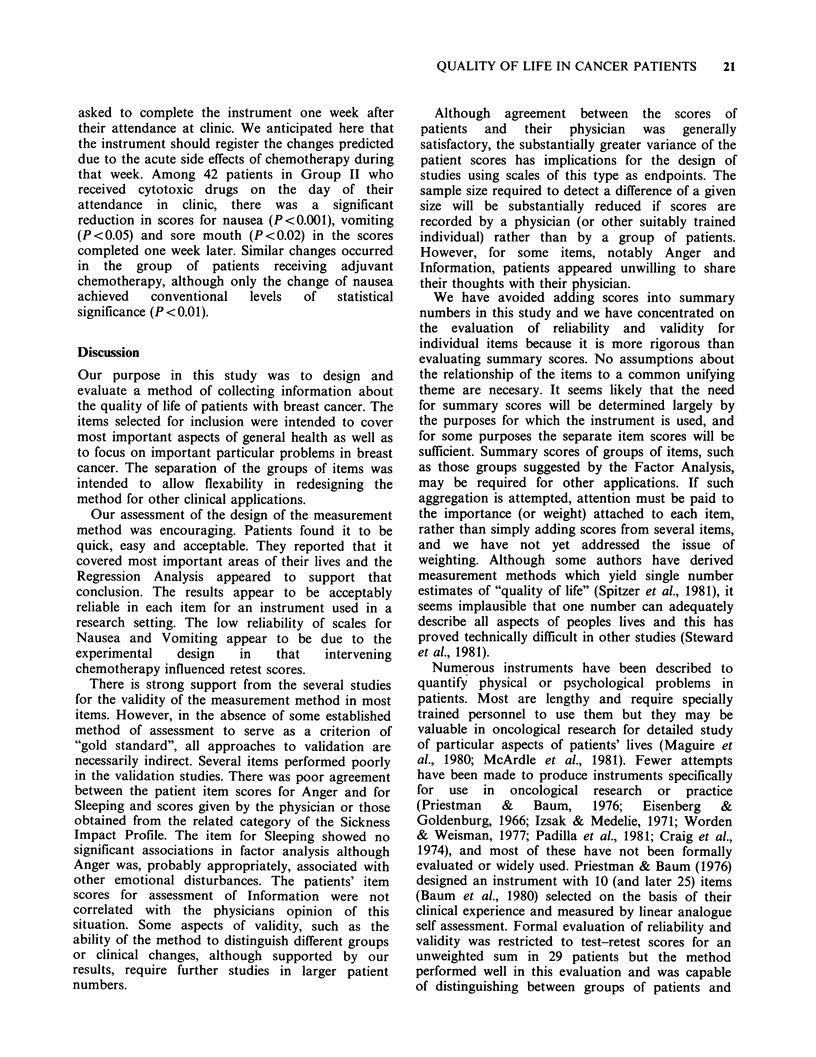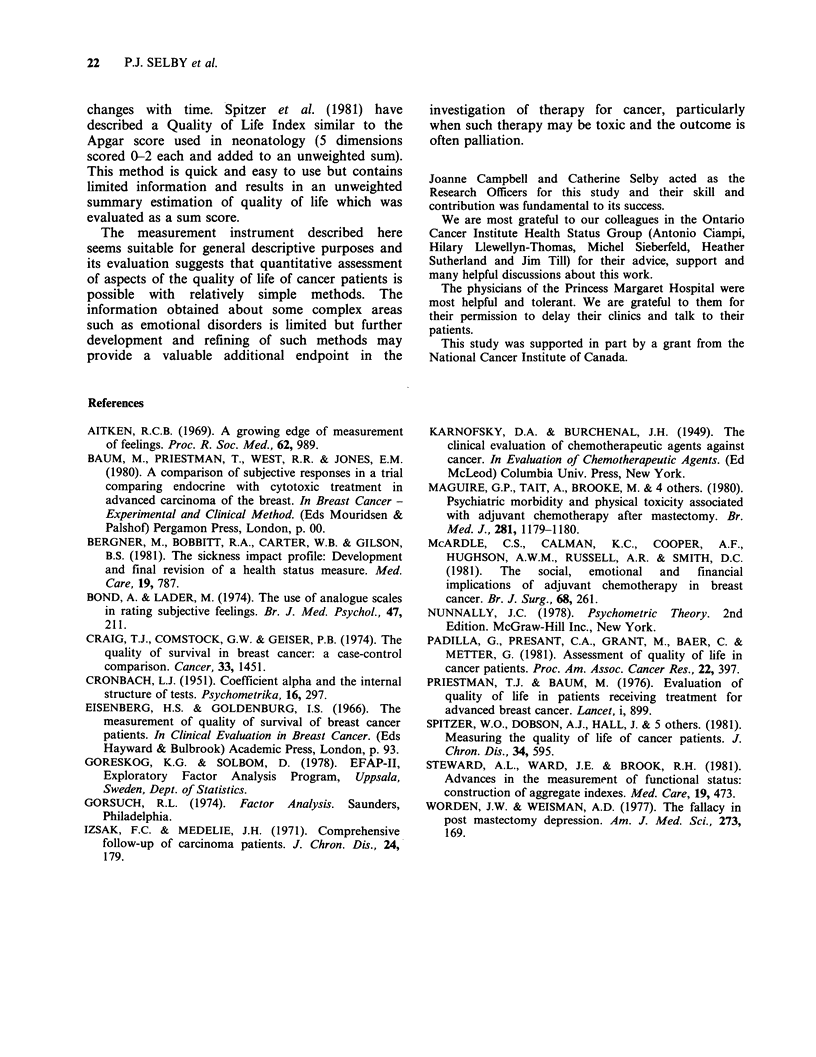# The development of a method for assessing the quality of life of cancer patients.

**DOI:** 10.1038/bjc.1984.134

**Published:** 1984-07

**Authors:** P. J. Selby, J. A. Chapman, J. Etazadi-Amoli, D. Dalley, N. F. Boyd

## Abstract

Although the need for a method of measuring the quality of life of patients undergoing therapy for cancer has been widely recognised, no adequately evaluated or feasible method has been established. We describe a method in which 31 items were assessed by patient self report using linear analogue scales. Eighteen items inquiring about general health problems were derived from the Sickness Impact Profile, an established method of assessing the effect of health upon behaviour and function. Thirteen items inquiring about major problems associated with breast cancer were derived from clinical experience and the opinions of patients with this disease. Each item of the measurement method (instrument) has been evaluated for content, feasibility, reliability and validity by methods that are widely used in psychometry but less familiar in medicine. It appeared easy to use, acceptable and reliable in these assessment. Validity was evaluated indirectly since no standard measurements of quality of life exist for comparison. Most items appeared valid when compared to alternative measurement methods including the Sickness Impact Profile and evaluation by a physician in a structured interview. The correlations between items in the instrument were analysed by factor analysis and seemed to fit with the clinical features of breast cancer. The method distinguished between clinically distinct groups of patients and detected changes with time. The study illustrates the possible approaches to the scientific evaluation of methods for measuring subjective features of patients lives. This method appears suitable for some purposes to measure quality of life in breast cancer and is intended to be flexible enough to be modified for other diseases. However, further evaluation, development and refinement will be needed before routine clinical application can be recommended.


Br. J. Cancer (1984), 50, 13–22

### The development of a method for assessing the quality of life of cancer patients

P.J. Selby*, J.-A.W. Chapman, J. Etazadi-Amoli, D. Dalley & N.F. Boyd

*Departments of Medicine and Bioresearch, Princess Margaret Hospital and Ontario Cancer Institute, Toronto, Ontario, Canada*

**Summary** Although the need for a method of measuring the quality of life of patients undergoing therapy for cancer has been widely recognised, no adequately evaluated or feasible method has been established. We describe a method in which 31 items were assessed by patient self report using linear analogue scales. Eighteen items inquiring about general health problems were derived from the Sickness Impact Profile, an established method of assessing the effect of health upon behaviour and function. Thirteen items inquiring about major problems associated with breast cancer were derived from clinical experience and the opinions of patients with this disease.

Each item of the measurement method (instrument) has been evaluated for content, feasability, reliability and validity by methods that are widely used in psychometry but less familiar in medicine. It appeared easy to use, acceptable and reliable in these assessment. Validity was evaluated indirectly since no standard measurements of quality of life exist for comparison. Most items appeared valid when compared to alternative measurement methods including the Sickness Impact Profile and evaluation by a physician in a structured interview. The correlations between items in the instrument were analysed by factor analysis and seemed to fit with the clinical features of breast cancer. The method distinguished between clinically distinct groups of patients and detected changes with time.

The study illustrates the possible approaches to the scientific evaluation of methods for measuring subjective features of patients lives. This method appears suitable for some purposes to measure quality of life in breast cancer and is intended to be flexible enough to be modified for other diseases. However, further evaluation, development and refinement will be needed before routine clinical application can be recommended.

When assessing the benefits of a treatment for a potentially disabling or fatal illness, we need to know about survival and the quality of survival. In cancer therapy, tumour volume changes, measures of treatment toxicity, patient performance status or disease-free interval may give valuable information. However, measurement of the quality of life of surviving patients is not at present possible because there is no adequately evaluated and feasible method for this purpose. The need for such a method is widely recognised. Its successful applications might include the identification of damaging effects of disease or treatment which could be reduced by changes in therapy. It may be possible to compare the effects of alternative treatments on both the quality and duration of survival. The provision of a more complete description of the effects of treatments might allow patients and physicians to choose more easily between alternatives.

Several problems must be addressed before measurement of the quality of life of patients can be used for these purposes. The first group of problems concerns the design of a method of measurement, and the second, the evaluation of the method before it is used in the clinical setting. In designing a method, decisions must be made about its scope, detail, whether data will be obtained from patients self-report or from others such as interviewers or physicians, and whether qualitative descriptions or quantitative measurements should be sought. The selection of an appropriate scope of inquiry is particularly important. Restriction of measurements to symptoms alone may fail to assess the impact of the disease on the patients whole life. On the other hand inquiries which attempt to be comprehensive (global) may fail to assess adequately a dominant symptom or preoccupation and tend to be lengthy and time-consuming. When the design problems are resolved, the method must be evaluated to find out if it meets the standards of other measurements used in medicine. Is it easy to use in a clinical setting and how large are the errors associated with the measurement (i.e. how reliable* is it)? Does it measure what it is intended to measure (i.e. how valid* is it)?

In this paper we describe an attempt to address these issues in the development of a method of

*Present address and address for correspondence: Section of Medicine, Institute of Cancer Research, Royal Marsden Hospital, Downs Road, Sutton, Surrey, UK.

Received 23 January 1984; accepted 26 March 1984.

© The Macmillan Press Ltd., 1984

measurement (a measurement "instrument"*) for the perceived quality of life of cancer patients. Data were collected by patients self assessment using linear analogue scales which are quite widely used in psychology but only recently applied in clinical oncology (Aitken, 1969; Bond & Lader, 1974; Priestman & Baum, 1976).

Two groups of items were chosen to define the scope of the method. Firstly, a global group were drawn from an existing comprehensive health index, the Sickness Impact Profile (SIP) (Bergner *et al.*, 1981). The second group of items were selected to assess important areas related to the disease under study. We chose to study breast cancer because of its frequency and because we believe that measurement of quality of life may be particularly important when the outcome of treatment is often palliation and treatments may be unpleasant.

### Methods and results

### *Patient population*

All patients attending a clinic for the medical management of breast cancer at the Princess Margaret Hospital between March 1981 and February 1982 were asked to take part in this study if they were able to understand spoken and written English. We attempted to include all eligible patients seen during the period of the study. Patients were asked to take part in these studies on 275 occasions and did so on 246 occasions. Patients refused on 29 occasions, often for reasons unrelated to the study, such as language difficulties (5 patients), recent bereavement (2 patients), and lost spectacles (1 patient). However, a few patients were unwilling to take part in studies of this type, including two who said that the questions made them more aware of their disability, three who said that too much time was required, and two others who wished to discuss their health only with their own physician.

Five groups of patients, each of which were accrued separately, were studied.

I 31 patients with either recurrent disease (16) or receiving adjuvant therapy (15). Mean age 51 years.
II 96 patients with recurrent disease aged < 70 years. 52 receiving chemotherapy. Mean age 57 years.
III 50 patients with recurrent disease. 27 receiving chemotherapy. Mean age 58 years.
IV 31 patients with recurrent disease. 13 receiving chemotherapy. Mean age 55 years.
V 23 patients receiving adjuvant chemotherapy for lymph node positive breast cancer surgically resected. Mean age 48 years.

### *Design of the self assessment method*

*Selection of items* The Sickness Impact Profile contains 136 questions grouped into 12 categories. It is based upon descriptions provided by a large and heterogeneous group of people about the way their behaviour and functions had been affected by illness. Each of the 12 categories can be scored separately to provide a quantitative index of severity, and in addition, a weighted overall score, which summarize the scores of all categories, can be generated (Bergner *et al.*, 1981). Five of the 12 categories in SIP (Work, Home Management, Recreation and Pastimes: Mobility, and Alertness Behaviour) were each represented by one linear analogue scale in our self assessment instrument. Two categories (Eating, Sleep and Rest) were each represented by 2 linear analogue scales to allow scoring of an increase or decrease in the activity. Five categories in SIP were represented by more than one linear analogue scale. The category, Social Interaction, in SIP was represented by separate scales for Social Life and Family Relationships, the SIP category of Body Care and Movement was represented by separate scales for Self-care and Physical Activity. The SIP category for Ambulation was represented also by this scale for Physical Activity. The SIP category of Emotional Behaviour was represented by separate scales for Anxiety, Depression, and Anger. The SIP category for Communication was represented by scales for Speech and for Writing.

The 12 categories of SIP thus gave rise to a total of 18 linear analogue scales that enquired about different aspects of general health.

In addition, we selected 12 items on the basis of clinical experience that described clinical problems or side effects of treatment that are common in this patient population. These were Pain, Breathing, Sore Mouth, Nausea, Vomiting, Hair Loss, Attractiveness, Appearance, Dysuria, Constipation, Diarrhoea and Fatigue. Each of these items was represented by one linear analogue scale.

Finally, we included an item related to overall quality of life referred to as the Uniscale (Figure 1) giving a total of 31 scales.

*Design of items* Each item was represented in the self assessment instrument by a title and a 10 cm linear analogue scale. Each scale was anchored at its ends by descriptive phrases, the right hand end

*The word "instrument" is widely used in psychology and psychiatry to denote a structured questionnaire which is subjected to formal assessment. Reliability is defined as the amount of random error in a measurement-error variance. Validity means the extent to which a measurement appropriately measures that which it is intended to measure.

**Figure 1**

The overall score (this scale) completed by patients and physicians.

PLEASE SCORE HOW YOU FEEL YOUR LIFE HAS BEEN AFFECTED BY THE STATE OF YOUR HEALTH (ANY DISEASE OR TREATMENT) DURING TODAY (24 h).

You may like to look back over the previous scales and consider the scores you have made and how much you feel they have affected your life.

| | | |
|---|---|---|
| My life is extremely unpleasant because of the state of my health | ______________________________ | My life is normal for me with no changes because of the state of my health |

**Figure 2**

Linear analogue self assessment scales – three examples completed by patients and physicians

PLEASE SCORE HOW YOU FEEL EACH OF THESE ASPECTS OF YOUR LIFE WAS AFFECTED BY THE STATE OF YOUR HEALTH DURING TODAY (24 h)

2. Nausea

| | | |
|---|---|---|
| extremely severe nausea | ______________________________ | no nausea |

13. Physical Activity

| | | |
|---|---|---|
| completely unable to move my body | ______________________________ | normal physical activity for me |

20. Depression

| | | |
|---|---|---|
| extremely depressed | ______________________________ | not depressed at all |

of the line describing normality or the absence of a symptom and the left hand end the opposite extreme of the state. Examples are shown in Figure 1. Items were randomly ordered to reduce the chance of scores on one item influencing scores on related adjacent items.

The patients were asked to place a vertical mark on each linear analogue scale in a position that they felt best described their own state with regard to that item. They were asked to consider the previous 24 h in some studies and previous 7 days in others (Table I). These instructions and the methods to be used were explained to patients in a standard way by one of two research assistants who answered any questions from patients before the first completion of the self assessment instrument.

To score patients' responses, each linear analogue scale was assigned a value 10 at the end indicating normality or the absence of a symptom, and 0 at the other end. The line was then measured in millimeters from 0. Higher scores thus indicated better health or less severe symptoms.

*Verification of content* (*regression analysis*) We assessed the relevance and importance of the selected items to patients with breast cancer by inviting comments from 31 patients (Group I) in an open questionnaire and by direct questioning a further 30 patients. These enquiries indicated that the items included were relevant and important to patients with this disease and elicited the suggestion that one item be added asking about satisfaction

**Table I** Summary of studies

| *Property evaluated* | *Method of evaluation* | *Patient group (No.)* | *Duration of enquiry (days)* | *Analytic method* |
|---|---|---|---|---|
| Feasibility | Time to complete | I (31) | 1 | — |
| | Acceptance rate | All (275) | 1 or 7 | — |
| Item selection | Patients opinion | I (31) | 1 | — |
| | Regression analysis | II (96) | 7 | Multiple regression |
| Reliability | Test–retest comparison | II (96) | 7 | Correlation |
| | | I (31) | 1 | Correlation |
| | Split half reliability | II (96) | 7 | Cronbach's alpha |
| Validity | Relationship between scores for different items | II (96) | 7 | Factor analysis |
| | Comparison to Sickness Impact Profile scores | III (50) | 1 | Correlation |
| | Comparison to Karnofsky Index scores | III (50) | 1 | Correlation |
| | Comparison to physician scores | III (50) | 1 | Correlation |
| | Distinguish between groups | II, V (75) | 7 | Comparison of means ($t$ test) |
| | Distinguish change over time | II, V (65) | 7 | Comparison of means (paired $t$ test) |

with information-provided about the disease and its treatment. This was done to give a final total of 32 scales (including the Uniscale).

In order to investigate further how completely the instrument might describe the quality of life of our patients, we examined the relationship between the 31 items each describing an aspect of their lives and the Uniscale which set out to provide a single overall score for quality of life. We made the hypothesis that the extent to which the variation in the Uniscale score was explained by the variation in the 31 items might be used as an indication of the completeness of item selection. We used the 96 patients' scores from Group II who completed the instrument on three occasions (twice for test–retest comparison and once a week later), and we determined the extent to which variation in Uniscale scores could be explained by variation in the scores of individual items using a multiple regression analysis. The patients' self-assessed quality of life score on the Uniscale was treated as the dependent variable in this analysis, and the scores of the remaining 31 items were treated as independent variables.

Variation in the scores for items was able to explain between 68% and 83% of the variation in Uniscale. Most of the variation in Uniscale scores, in each of the three analyses, was explained by variation in scores of Physical Activity, although scores for Depression also made a significant contribution. Other variables including Social Life, Anxiety and Appearance made significant contributions to one or more of the three analyses, but not to all of them.

This result suggests that the items selected do make a major contribution to the patients' perceived quality of life. However, this result should be interpreted with caution because Uniscale was completed by patients immediately after completing the other scales and the independence of the two measures is thus not assured.

*Statistical procedures*

Scores for all items had a unimodal distribution that was highly skewed toward the end of the scale indicating normality or the absence of a symptom. The influence of this distribution upon the statistical analyses was examined in two ways. First, we performed all analyses before and after the exclusion of scores that indicated normality. This was done by arbitrarily selecting a cut-off point at 9.5, and excluding all scores above this point. Second, all analyses were carried before and after transformation of the data to obtain a more normal distribution. Several methods of transformation were examined for this purpose including log transformation, arc–sin transformation, and arc–cosine transformations. Arc–cosine transformation resulted in the most normal distribution of values and was the procedure adopted by us.

The statistical tests employed are listed in Table I. Coefficients of agreement (intra-class correlation coefficients), Pearson product-moment correlation coefficients, and t-tests were carried out using the Statistical Packages for the Social Sciences (SPSS), Update 7–9. Factor analysis was done using the Exploratory Factor Analysis Program (Goreskog & Solbom, 1978). The Multiple Regression Analysis was performed using SPSS computer programmes.

No generally accepted rules exist for the degree of correlation which is required to support or refute the reliability or validity of a test. In this study we have followed the general recommendations of Nunnally (1978) for a test used in a research setting. These probably represent quite rigorous criteria for the assessment of individual items within the instrument. Summation of item scores would lead to higher reliability or validity estimations. Coefficients of correlation are regarded as strongly supporting reliability when greater than 0.7. Validity estimations are constrained at their upper limit by the reliability of the item and coefficients greater than 0.6 strongly argue for validity. Reliability coefficients are expected not to be lower than validity measurements on theoretical grounds. For readers who prefer to evaluate the correlation coefficients by estimating their statistical significance, we have given $P$ values in footnotes to the Tables. In all cases, correlation coefficients of more than 0.6 were significant at the $P<0.01$ level.

### *Evaluation of the self assessment method*

We evaluated the feasibility of the instrument in the clinical setting as well as the reliability and validity of the resulting scores using methods that are summarized in Table I. The results are described for each objective rather than for each separate study.

*Feasibility* The 31 patients in Group I each completed the instrument on 4 occasions. Mean completion time was 3.6 min (±2.6 min, s.d).

*Reliability* The reliability of each scale was assessed by asking the 96 patients in Group II to complete the instrument on 2 occasions. The first of these was in the morning in the Outpatient Clinic, and the second 9–12 h later at home. Test-retest reliability was assessed by comparing the scores recorded on these two occasions. We anticipated that the patient's clinical state would not change between the first and second completions of the instrument, but some patients in this group did receive chemotherapy between test and retest. Table II shows the coefficients of agreement between the scores completed on two occasions. Sixteen of the 18 scales for general health items gave correlations of agreement greater than 0.70 and the remaining two were between 0.60 and 0.70. Seven of the 13 scales for disease or treatment related items were also above 0.70 and three were between 0.60 and 0.70. The coefficient of agreement for Uniscale, the global quality of life scale, was 0.72.

The scales for nausea, vomiting and diarrhoea appear less reliable in this setting, with coefficients of agreement less than 0.40. To examine the possibility that the reliability of scores for these symptoms had been influenced by the administration of chemotherapy between the first and second completions of the instrument, we recalculated coefficients of agreement after excluding patients who received chemotherapy. Higher coefficients of agreement were observed (0.40 for nausea and 0.30 for Vomiting) but they remained lower than for the other items. The suggestion that these low scores may be explained by the intervening chemotherapy is supported by the

**Table II** Test–retest reliability of items

| *General Health item* | | | | *Disease related item* | | | |
|---|---|---|---|---|---|---|---|
| *Item* | *r* | *Item* | *r* | *Item* | *r* | *Item* | *r* |
| Work | 1.00 | Social life | 0.78 | Dysuria | 0.85 | Sore mouth | 0.68 |
| Increased | | Housework | 0.75 | Attractiveness | 0.84 | Breathing | 0.66 |
| eating | 0.96 | Reduced | | Pain | 0.83 | Fatigue | 0.66 |
| Writing | 0.92 | eating | 0.74 | Information | 0.79 | | |
| Anger | 0.90 | Physical | | Constipation | 0.79 | Diarrhoea | 0.37 |
| Reduced | | activity | 0.72 | Hair loss | 0.78 | Nausea | 0.32 |
| sleep | 0.82 | Family | | Appearance | 0.78 | Vomiting | 0.25 |
| Concentration | 0.81 | relations | 0.70 | | | | |
| Self care | 0.81 | Anxiety | 0.70 | | | | |
| Depression | 0.80 | | | | | Uniscale | 0.72 |
| Increased | | | | | | | |
| sleep | 0.79 | Mobility | 0.64 | | | | |
| | | Speech | 0.63 | | | | |
| Recreation | 0.78 | | | | | | |

Correlation coefficients are highly significant, $P<0.001$, except those for Diarrhoea ($P=0.005$), Nausea ($P=0.002$) and Vomiting ($P=0.012$).

findings of a small test–retest reliability study of the 31 patients in Group I who completed the instrument in the evening before clinic and again on arrival in the clinic. These results are not shown in full. However, the test–retest correlation coefficient for the scale for Nausea and Vomiting in this study was greater than 0.7.

The reliability of the whole instrument was further examined by comparing scores in 2 halves for the 96 patients in Group II ("split-half reliability") using methods described by Cronbach (1951). Cronbach's statistic Alpha gave a value of 0.91. This statistic is widely used for evaluating measurement instruments and this value supports the general impression of reliability here. However there are theoretical objections to its use for this purpose (Cronbach, 1951) and it must be interpreted cautiously.

To examine the influence of scores that indicated no impairment for an item upon the assessment of reliability, all of the data shown were analysed before and after the exclusion of scores above 9.5. Further analyses were carried out after arc–cosine transformation of scores to assess the effect upon the results of data that were not normally distributed. In both instances, co-efficients of agreement were obtained that were very similar to those shown in Table II and the general conclusions presented in that Table were not altered by these additional analyses.

*Validity* Validity, the extent to which scores truly describe the severity of the state being assessed, is the most difficult aspect of evaluation because there is no accepted alternative method of measurement to serve as a criterion against which the present instrument can be judged. In the absence of such a criterion, we adopted four indirect methods for evaluating validity.

1. Correlations between scores for items within the Instrument (Factor Analysis).

The extent to which scores on individual items correlated with scores on other related items was assessed. Thus, it is expected that patients with severe pain or extreme difficulty in breathing will also be restricted in physical activity and, if the item is a true measure of the patients' state, that the scores on these items will be correlated with each other. The relationship between the item scores by the 96 patients in Group II was assessed using factor analysis (Gorsuch, 1974). This computer-based technique examines correlations between scores on all items and creates groups of items whose scores are most strongly correlated with each other. If scores are valid it is expected that the groups created by factor analysis will be comprised of items that are expected, on clinical or other grounds, to be associated with each other.

The results are shown in Table III. The item "Work" was omitted from this analysis because of the large number of patients who did not normally work outside the home. The items for Mouth Soreness, Dysuria, Speech and Self Care had a very narrow distribution in the upper part of the range (means >9.5) and their variance was too small to allow factor analysis. The item for Sleep did not correlate significantly with any factor.

Table III shows the five groups of items, or "factors", that were generated by this analysis. Each factor is comprised of items whose scores were strongly correlated with that factor. The factor "loadings", which are shown in the Table in parenthesis, are a measure of the strength of this association, and can be considered similar to a correlation coefficient. The items of Physical Activity, Concentration, and Family Relations were each significantly associated with two factors.

Factor analysis yields groups of items which can

**Table III** Correlations between items: factor analysis

| *Factor 1* | | *Factor 2* | | *Factor 3* | | *Factor 4* | | *Factor 5* | |
|---|---|---|---|---|---|---|---|---|---|
| Housework | (0.89) | Pain | (0.60) | Depression | (0.80) | Nausea | (0.86) | Attractiveness | (0.64) |
| Recreation | (0.85) | Physical activity | (0.57) | Anger | (0.77) | Vomiting | (0.85) | Family relations | (0.63) |
| Social life | (0.71) | Bowel habit | (0.54) | Anxiety | (0.68) | Eating | (0.38) | Hair loss | (0.35) |
| Mobility | (0.49) | Breathing | (0.42) | Appearance | (0.40) | | | | |
| Fatigue | (0.46) | | | Concentration | (0.40) | | | | |
| Writing | (0.44) | | | Family relations | (0.38) | | | | |
| Physical activity | (0.42) | | | | | | | | |
| Concentration | (0.40) | | | | | | | | |

Figures in brackets are rotated factor loading. All loadings >0.3 are shown.

be said to be associated on statistical grounds. The analysis will only support the validity of the instrument if the factorial associations appear on independent grounds to be biologically or clinically real. It is the authors' opinion, based on their experience of the clinical features of metastatic breast cancer, that the factors in general show the relationships expected in this disease. Factors 1 and 2 reveal the relationship between impaired physical, recreational, social and working activities and the common symptoms – fatigue, pain and breathlessness – that are responsible for functional impairment in breast cancer. The association of pain with altered bowel habit may well be explained by the constipating effect of analgesics. Factor 3 expressed expected associations between different emotional disturbances and their effect on concentration and family relations. The impact of physical appearance on emotional state in this disease is widely recognised. Factor 4 represents alimentary disturbance by disease and treatment. Factor 5 shows the effect of hair loss on the perception on attractiveness which might be expected to influence relationships with other family members, particularly spouses.

The factor analysis does appear to show that clinically related items are associated with each other and hence supports the validity of the measurement method.

2. Comparison with scores obtained by alternative methods (the Sickness Impact Profile, Physician Interviews and Karnofsky scores).

The group of items intended to measure general health features, derived from the Sickness Impact Profile, was evaluated by comparison of linear analogue scores to category scores in SIP in the 50 patients in Group III. All of the scores for self assessment on the linear analogue scales by this group of patients were compared to scores made by a physician on a linear analogue scale. The physician was not involved in the patients care and his interview was carried out in a structured way. The first part consisted of an open interview in which the patients were encouraged to describe their symptoms and related problems. The second part consisted of specific questioning about each item of the questionnaire. The Uniscale scores were compared to a Uniscale score given by the physician, to the weighted sum given by the Sickness Impact Profile and to scores on the Karnofsky Performance Index (Karnofsky & Burchenal, 1949).

(i) The Sickness Impact Profile

Table IV shows the correlation coefficients observed when scores obtained from the 18 items related to general health were compared with the scores derived from the associated categories of the Sickness Impact Profile.

**Table IV** Correlation of item scores with Sickness Impact Profile categories

| *Direct comparisons* | | *Indirect comparisons* | |
|---|---|---|---|
| *Item* | *r* | *Item* | *r* |
| Work | 0.97 | Depression | 0.98 |
| Housework | 0.71 | Self care | 0.74 |
| Overall score | 0.70 | Social life | 0.71 |
| | | Writing | 0.62 |
| Physical activity | 0.65 | Speech | 0.48 |
| | | Anxiety | 0.48 |
| Reduced eating | 0.64 | | |
| Mobility | 0.62 | Family relations | 0.41 |
| Recreation | 0.47 | | |
| | | Sleep | 0.38 |
| Concentration | 0.47 | Anger | 0.28 |

Coefficients significant ($P<0.001$) except those for Family relations ($P<0.005$), Sleep and Anger ($P<0.05$).

Because of the way in which items were selected (see Selection of items) some did not directly correspond to a category in the Sickness Impact Profile. For example, the Sickness Impact Profile does not generate separate scores for Anxiety and Depression although questions concerning each of these symptom complexes are contained in the Sickness Impact Profile category, "Emotional Behaviour". We therefore compared the item scores for Anxiety and Depression for the self assessment instrument with the Sickness Impact Profile category of Emotional Behaviour. Indirect relationships of this type are listed separately in Table IV.

All correlation coefficients between items in the self assessment instrument and the Sickness Impact Profile were statistically significant ($P<0.001$). As expected, correlations were strongest when there was a direct correspondence between an item and a Sickness Impact Profile category, where 7 of 8 comparisons gave correlation coefficients greater than 0.60. For the 9 items where there was only an indirect relationship between the compared scores, 4 correlation coefficients were greater than 0.60, 3 between 0.40 and 0.50 and 2 were less than 0.40.

These correlations were again carried out before and after the exclusion of scores greater than 9.5, and after arc–cosine transformation of scores, with results that remained very similar to those shown in Table IV.

(ii) Physician Interviews

Before comparing the linear analogue scores of patients and a physician, we first assessed the reliability of linear analogue scoring by a physician by comparing the scores assigned by two of us (PS and NFB) who independently interviewed a series of 30 patients with metastatic breast cancer (Group

IV). The comparison of these scores showed that the coefficients of agreement on scores between two physicians were similar to test–retest reliability when patients completed the instrument.

Table V shows the correlation coefficients obtained when the scores assigned by patients were compared with those assigned by the physician. Eleven of the 18 general health items have correlation coefficients greater than 0.70 and a further 3 were between 0.60 and 0.70. Six of the 12 disease or treatment related items had correlation coefficients greater than 0.70, and 3 were between 0.60 and 0.70.

Correlation coefficients were less than 0.5 for six items: Increased Sleep, Speech and Anger from the general health group; Sore Mouth, Information and Dysuria from the disease-related group. Closer inspection of the data shows that both patients and physician scores were high (mean value >9.5) with small variance for the items Speech, Sore Mouth and Dysuria so that substantial agreement existed which is not reflected in the correlation coefficient because of lack of dispersal of the data. However, scores were adequately dispersed for the items Anger, Information and Increased Sleeping and the low correlation coefficients indicate poor agreement.

Although the scores of patients and the physician were, for the most part, strongly correlated, the variances associated with these scores differed systematically. The variance of the linear analogue scores assigned by a physician were often 1/2 and sometimes 1/5 that of the scores for the patients themselves. This finding is not unexpected, because the variances in the patients' scores arise both from differences between individuals (i.e. from differences in the severity of symptoms between individuals) and from differences in the error associated with measurement and the latter source of variation would be expected to a substantially reduced in the measurements made by a single trained individual.

(iii) Overall scores

The Uniscale scores completed by the patient and by the physician together with the physician scores for the Karnofsky Index and the overall weighted sum deduced from the Sickness Impact Profile might be regarded as single figures intended to give an overall indication of the patients' well being. Coefficients of agreement between these were examined and they were all highly significantly ($P<0.001$) intercorrelated. The patients Uniscale score correlated closely with the physicians Uniscale score and the SIP overall score ($r>0.7$) and moderately with the Karnofsky Indexes ($r>0.6$).

3. Distinction between groups of patients

The scores of patients with metastatic breast cancer in Group II were significantly lower, indicating greater impairment, than those of patients receiving adjuvant chemotherapy in Group V for the items of Mobility, Breathing, Pain, Physical Activity, Housework, Writing and Reduced Eating. Patients receiving adjuvant therapy scored significantly lower for the items of Hair Loss, Attractiveness, and Increased Eating. Scores for Hair Loss and Attractiveness remained significantly lower in the adjuvant group even when the comparison was confined to patients with metastatic disease who are receiving identical chemotherapy. The instrument was therefore able to distinguish clinically different groups of patients.

4. Detection of change with time

Patients with metastatic breast cancer in Group II and patients receiving adjuvant chemotherapy, were

**Table V** Correlation of scores from self assessment and assessment by a physician

| *General health item* | | | | *Disease related item* | | | |
|---|---|---|---|---|---|---|---|
| *Item* | *r* | *Item* | *r* | *Item* | *r* | *Item* | *r* |
| Writing | 0.88 | Family relations | 0.70 | Diarrhoea | 0.98 | Nausea | 0.62 |
| Mobility | 0.78 | Housework | 0.69 | Constipation | 0.89 | Appearance | 0.57 |
| Self care | 0.77 | Concentration | 0.66 | Hair loss | 0.80 | Sore mouth | 0.40 |
| Physical activity | 0.76 | Anxiety | 0.60 | Breathing | 0.80 | Information | −0.09 |
| Recreation | 0.76 | Depression | 0.58 | Fatigue | 0.74 | Dysuria | −0.04 |
| Social life | 0.75 | Increased sleep | 0.37 | Pain | 0.72 | | |
| Reduced eating | 0.75 | Speech | 0.29 | Attractiveness | 0.66 | | |
| Reduced sleep | 0.75 | Anger | 0.11 | Vomiting | 0.64 | | |
| Work | 0.75 | | | | | | |
| Increased eating | 0.71 | | | | | | |

Coefficients significant ($P<0.001$) except those for Increased Sleep, Sore Mouth and Speech ($P<0.05$), Anger, Information and Dysuria ($P<0.1$).

asked to complete the instrument one week after their attendance at clinic. We anticipated here that the instrument should register the changes predicted due to the acute side effects of chemotherapy during that week. Among 42 patients in Group II who received cytotoxic drugs on the day of their attendance in clinic, there was a significant reduction in scores for nausea ($P<0.001$), vomiting ($P<0.05$) and sore mouth ($P<0.02$) in the scores completed one week later. Similar changes occurred in the group of patients receiving adjuvant chemotherapy, although only the change of nausea achieved conventional levels of statistical significance ($P<0.01$).

### Discussion

Our purpose in this study was to design and evaluate a method of collecting information about the quality of life of patients with breast cancer. The items selected for inclusion were intended to cover most important aspects of general health as well as to focus on important particular problems in breast cancer. The separation of the groups of items was intended to allow flexability in redesigning the method for other clinical applications.

Our assessment of the design of the measurement method was encouraging. Patients found it to be quick, easy and acceptable. They reported that it covered most important areas of their lives and the Regression Analysis appeared to support that conclusion. The results appear to be acceptably reliable in each item for an instrument used in a research setting. The low reliability of scales for Nausea and Vomiting appear to be due to the experimental design in that intervening chemotherapy influenced retest scores.

There is strong support from the several studies for the validity of the measurement method in most items. However, in the absence of some established method of assessment to serve as a criterion of "gold standard", all approaches to validation are necessarily indirect. Several items performed poorly in the validation studies. There was poor agreement between the patient item scores for Anger and for Sleeping and scores given by the physician or those obtained from the related category of the Sickness Impact Profile. The item for Sleeping showed no significant associations in factor analysis although Anger was, probably appropriately, associated with other emotional disturbances. The patients' item scores for assessment of Information were not correlated with the physicians opinion of this situation. Some aspects of validity, such as the ability of the method to distinguish different groups or clinical changes, although supported by our results, require further studies in larger patient numbers.

Although agreement between the scores of patients and their physician was generally satisfactory, the substantially greater variance of the patient scores has implications for the design of studies using scales of this type as endpoints. The sample size required to detect a difference of a given size will be substantially reduced if scores are recorded by a physician (or other suitably trained individual) rather than by a group of patients. However, for some items, notably Anger and Information, patients appeared unwilling to share their thoughts with their physician.

We have avoided adding scores into summary numbers in this study and we have concentrated on the evaluation of reliability and validity for individual items because it is more rigorous than evaluating summary scores. No assumptions about the relationship of the items to a common unifying theme are necesary. It seems likely that the need for summary scores will be determined largely by the purposes for which the instrument is used, and for some purposes the separate item scores will be sufficient. Summary scores of groups of items, such as those groups suggested by the Factor Analysis, may be required for other applications. If such aggregation is attempted, attention must be paid to the importance (or weight) attached to each item, rather than simply adding scores from several items, and we have not yet addressed the issue of weighting. Although some authors have derived measurement methods which yield single number estimates of "quality of life" (Spitzer *et al.*, 1981), it seems implausible that one number can adequately describe all aspects of peoples lives and this has proved technically difficult in other studies (Steward *et al.*, 1981).

Numerous instruments have been described to quantify physical or psychological problems in patients. Most are lengthy and require specially trained personnel to use them but they may be valuable in oncological research for detailed study of particular aspects of patients' lives (Maguire *et al.*, 1980; McArdle *et al.*, 1981). Fewer attempts have been made to produce instruments specifically for use in oncological research or practice (Priestman & Baum, 1976; Eisenberg & Goldenburg, 1966; Izsak & Medelie, 1971; Worden & Weisman, 1977; Padilla *et al.*, 1981; Craig *et al.*, 1974), and most of these have not been formally evaluated or widely used. Priestman & Baum (1976) designed an instrument with 10 (and later 25) items (Baum *et al.*, 1980) selected on the basis of their clinical experience and measured by linear analogue self assessment. Formal evaluation of reliability and validity was restricted to test–retest scores for an unweighted sum in 29 patients but the method performed well in this evaluation and was capable of distinguishing between groups of patients and changes with time. Spitzer *et al.* (1981) have described a Quality of Life Index similar to the Apgar score used in neonatology (5 dimensions scored 0–2 each and added to an unweighted sum). This method is quick and easy to use but contains limited information and results in an unweighted summary estimation of quality of life which was evaluated as a sum score.

The measurement instrument described here seems suitable for general descriptive purposes and its evaluation suggests that quantitative assessment of aspects of the quality of life of cancer patients is possible with relatively simple methods. The information obtained about some complex areas such as emotional disorders is limited but further development and refining of such methods may provide a valuable additional endpoint in the investigation of therapy for cancer, particularly when such therapy may be toxic and the outcome is often palliation.

Joanne Campbell and Catherine Selby acted as the Research Officers for this study and their skill and contribution was fundamental to its success.

We are most grateful to our colleagues in the Ontario Cancer Institute Health Status Group (Antonio Ciampi, Hilary Llewellyn-Thomas, Michel Sieberfeld, Heather Sutherland and Jim Till) for their advice, support and many helpful discussions about this work.

The physicians of the Princess Margaret Hospital were most helpful and tolerant. We are grateful to them for their permission to delay their clinics and talk to their patients.

This study was supported in part by a grant from the National Cancer Institute of Canada.

### References

AITKEN, R.C.B. (1969). A growing edge of measurement of feelings. *Proc. R. Soc. Med.*, **62**, 989.

BAUM, M., PRIESTMAN, T., WEST, R.R. & JONES, E.M. (1980). A comparison of subjective responses in a trial comparing endocrine with cytotoxic treatment in advanced carcinoma of the breast. *In Breast Cancer – Experimental and Clinical Method.* (Eds Mouridsen & Palshof) Pergamon Press, London, p. 00.

BERGNER, M., BOBBITT, R.A., CARTER, W.B. & GILSON, B.S. (1981). The sickness impact profile: Development and final revision of a health status measure. *Med. Care*, **19**, 787.

BOND, A. & LADER, M. (1974). The use of analogue scales in rating subjective feelings. *Br. J. Med. Psychol.*, **47**, 211.

CRAIG, T.J., COMSTOCK, G.W. & GEISER, P.B. (1974). The quality of survival in breast cancer: a case-control comparison. *Cancer*, **33**, 1451.

CRONBACH, L.J. (1951). Coefficient alpha and the internal structure of tests. *Psychometrika*, **16**, 297.

EISENBERG, H.S. & GOLDENBURG, I.S. (1966). The measurement of quality of survival of breast cancer patients. *In Clinical Evaluation in Breast Cancer.* (Eds Hayward & Bulbrook) Academic Press, London, p. 93.

GORESKOG, K.G. & SOLBOM, D. (1978). EFAP-II, Exploratory Factor Analysis Program, *Uppsala, Sweden, Dept. of Statistics.*

GORSUCH, R.L. (1974). *Factor Analysis.* Saunders, Philadelphia.

IZSAK, F.C. & MEDELIE, J.H. (1971). Comprehensive follow-up of carcinoma patients. *J. Chron. Dis.*, **24**, 179.

KARNOFSKY, D.A. & BURCHENAL, J.H. (1949). The clinical evaluation of chemotherapeutic agents against cancer. *In Evaluation of Chemotherapeutic Agents.* (Ed McLeod) Columbia Univ. Press, New York.

MAGUIRE, G.P., TAIT, A., BROOKE, M. & 4 others. (1980). Psychiatric morbidity and physical toxicity associated with adjuvant chemotherapy after mastectomy. *Br. Med. J.*, **281**, 1179–1180.

McARDLE, C.S., CALMAN, K.C., COOPER, A.F., HUGHSON, A.W.M., RUSSELL, A.R. & SMITH, D.C. (1981). The social, emotional and financial implications of adjuvant chemotherapy in breast cancer. *Br. J. Surg.*, **68**, 261.

NUNNALLY, J.C. (1978). *Psychometric Theory.* 2nd Edition. McGraw-Hill Inc., New York.

PADILLA, G., PRESANT, C.A., GRANT, M., BAER, C. & METTER, G. (1981). Assessment of quality of life in cancer patients. *Proc. Am. Assoc. Cancer Res.*, **22**, 397.

PRIESTMAN, T.J. & BAUM, M. (1976). Evaluation of quality of life in patients receiving treatment for advanced breast cancer. *Lancet*, i, 899.

SPITZER, W.O., DOBSON, A.J., HALL, J. & 5 others. (1981). Measuring the quality of life of cancer patients. *J. Chron. Dis.*, **34**, 595.

STEWARD, A.L., WARD, J.E. & BROOK, R.H. (1981). Advances in the measurement of functional status: construction of aggregate indexes. *Med. Care*, **19**, 473.

WORDEN, J.W. & WEISMAN, A.D. (1977). The fallacy in post mastectomy depression. *Am. J. Med. Sci.*, **273**, 169.